# Surgical Treatments for Lumbar Spine Diseases (TLIF vs. Other Surgical Techniques): A Systematic Review and Meta-Analysis

**DOI:** 10.3389/fsurg.2022.829469

**Published:** 2022-03-14

**Authors:** Kanthika Wasinpongwanich, Tanawin Nopsopon, Krit Pongpirul

**Affiliations:** ^1^Department of Orthopedics, Faculty of Medicine, Suranaree University of Technology, Nakhon Ratchasima, Thailand; ^2^Bumrungrad International Hospital, Bangkok, Thailand; ^3^Department of Preventive and Social Medicine, Faculty of Medicine, Chulalongkorn University, Bangkok, Thailand; ^4^Department of International Health, Johns Hopkins Bloomberg School of Public Health, Baltimore, MD, United States

**Keywords:** transforaminal lumbar interbody fusion, lumbar disease, meta-analysis, spondylolisthesis, spine fusion surgery

## Abstract

**Objective:**

The purpose of this study is to compare fusion rate, clinical outcomes, complications among transforaminal lumbar interbody fusion (TLIF), and other techniques for lumbar spine diseases.

**Design:**

This is a systematic review and meta-analysis.

**Data Sources:**

PubMed, EMBASE, Scopus, Web of Science, and CENTRAL databases were searched from January 2013 through December 2019.

**Eligibility Criteria for Selecting Studies:**

Randomized controlled trials (RCTs) that compare lumbar interbody fusion with posterolateral fusion (PLF) and/or other lumbar interbody fusion were included for the review.

**Data Extraction and Synthesis:**

Two independent reviewers extracted relevant data and assessed the risk of bias. Meta-analysis was performed using a random-effects model. Pooled risk ratio (RR) or mean difference (MD) with a 95% confidence interval of fusion rate, clinical outcomes, and complications in TLIF and other techniques for lumbar spinal diseases.

**Results:**

Of 3,682 potential studies, 15 RCTs (915 patients) were included in the meta-analysis. Compared to other surgical techniques, TLIF had slightly lower fusion rate [RR = 0.84 (95% CI = 0.72–0.97), *p* = 0.02, *I*^2^ = 0.0%] at 1-year follow-up whereas there was no difference on fusion rate at 2-year follow-up [RR = 1.06 (95% CI = 0.96–1.18), *p* = 0.27, *I*^2^ = 69.0%]. The estimated RR of total adverse events [RR = 0.90 (95% CI = 0.59–1.38), *p* = 0.63, *I*^2^ = 0.0%] was similar to no fusion, PLF, PLIF, and XLIF groups, and revision rate [RR = 0.78 (95% CI = 0.34–1.79), *p* = 0.56, *I*^2^ = 39.0%] was similar to PLF and XLIF groups. TLIF had approximately half an hour more operative time than other techniques (no fusion, ALIF, PLF, PLIF, XLIF) [MD = 31.88 (95% CI = 5.33–58.44), *p* = 0.02, *I*^2^ = 92.0%]. There was no significant difference between TLIF and other techniques in terms of blood loss (no fusion, PLIF, PLF) and clinical outcomes (PLF).

**Conclusions:**

Besides fusion rate at 1-year follow-up and operative time, TLIF has a similar fusion rate, clinical outcomes, parameters concerning operation and complications to no fusion, PLF, and other interbody fusion (PLIF, ALIF, XLIF).

**Systematic Review Registration:**

https://www.crd.york.ac.uk/prospero/, identifier: CRD42020186858.

## Introduction

Surgical treatment is mandatory in some patients with lumbar spine diseases. Whereas, cases without clinical or radiographic instability require only decompression, most lumbar spine diseases with instability especially the degenerative condition further proceed to spinal arthrodesis. The purpose of the treatment is to achieve solid fusion, correction of deformity, indirect nerve decompression, and stabilization. To obtain spine fusion, many operative techniques have been developed with different fusion rates and clinical results. The spinal fusion procedures could be categorized into posterior fusion (PF), posterolateral fusion (PLF), posterior lumbar interbody fusion (PLIF), transforaminal lumbar interbody fusion (TLIF), anterior lumbar interbody fusion (ALIF), extreme lateral interbody fusion (XLIF), the so-called lateral lumbar interbody fusion (LLIF), and oblique lumbar interbody fusion (OLIF). Total disc replacement (TDR) is an alternative option for patients to preserve spinal mobility.

Cloward et al. first described PLIF in 1952 (1) whereas Harm and Rollinger introduced TLIF three decades later (2). In early 2002, the minimally invasive surgical (MIS) approach was promoted to TLIF by Foley and Lefkowitz to improve peri–post-operative morbidity and clinical results (3). ALIF has a long history in the tuberculous spine; however, the technique was adapted to other lumbar spine diseases (4). Ozgur et al. describe a novel spine procedure called the XLIF in 2006 (5).

Several systematic reviews compared either MIS-TLIF or open TLIF with other fusion techniques, for example, MIS vs. open TLIF/PLIF (6), TLIF vs. ALIF (7), MIS-TLIF vs. LLIF (8), TLIF vs. PLIF (9), and TLIF vs. PLF (10). The studies were conducted from 2014 to 2020 (6–8, 10–13). Most of them compared only one or two techniques with TLIF for lumbar spine diseases (6–13). Half of them concluded that the level of evidence in their study was low and need more randomized controlled trials (RCTs) (6–8, 10, 14). The comparison of fusion rates, clinical outcomes, and complications among operative techniques for lumbar spine diseases has been inconclusive.

This systematic review and meta-analysis aimed to offer results based on fusion rate, clinical outcomes (VAS back and leg pain, ODI), parameters concerning operation and complications between TLIF, decompression alone (no fusion), PLF, and other interbody fusion (PLIF, ALIF, and XLIF).

## Methods

This study was conducted following the recommendations of the Preferred Reporting Items of Systematic Reviews and Meta-Analyses (PRISMA) statement. We prospectively registered the systematic review with PROSPERO International Prospective Register of Ongoing Systematic Reviews (registration number: CRD42020186858).

### Search Strategy

The PubMed, EMBASE, Scopus, Web of Science, and CENTRAL databases were searched for studies published between January 2010 and January 2019. The electronic databases were searched up to February 13, 2020. The reproducible search strategy was presented in detail in the Supplementary Material. Besides, the reference lists of included articles were searched, as well as related citations from other journals *via* Google Scholar.

### Study Selection

Only RCTs that compare lumbar interbody fusion with PLF and/or other lumbar interbody fusion were anticipated in this review. Inclusion criteria were established as follows: (1) the studies with a population of patients aged more than 18 years (2) RCT investigating lumbar spine disease treated with any lumbar interbody fusion or PLF or no fusion, (3) the study included at least one outcome (fusion rate, disability and pain or complications, operative time, blood loss, and hospital length of stay). Exclusion criteria were as follows: (1) biomechanical and cadaveric studies, (2) paper that is not in English, (3) duplicated studies.

**Figure 1:**
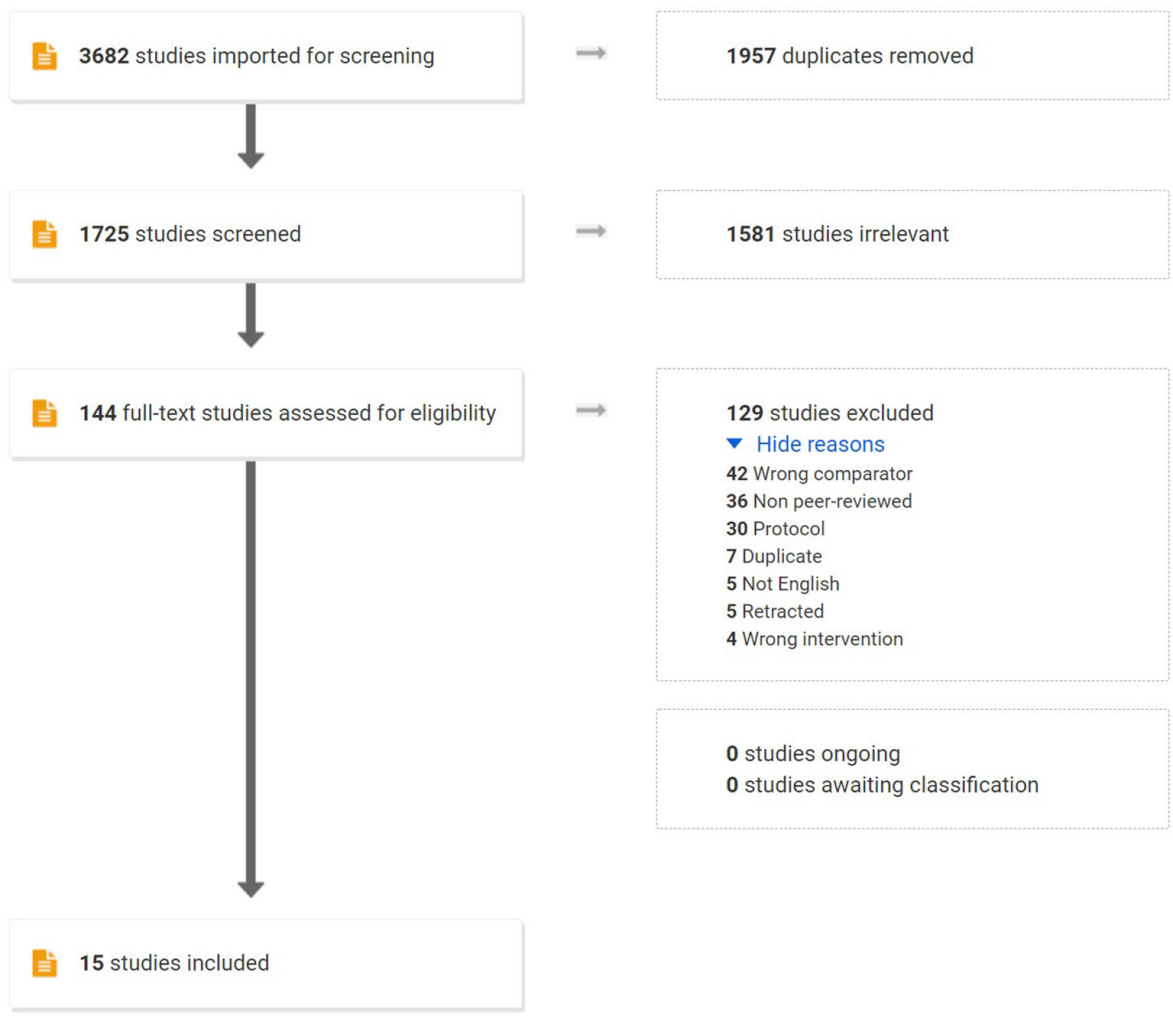
Flowchart of this systematic review with meta-analysis of prospective studies. RCTs, randomized controlled trials.

The title and abstracts of each study were independently reviewed by two authors (KW and TN) to assess for inclusion in the meta-analysis. For studies that meet the inclusion criteria, two reviewers (KW and TN) independently reviewed the full manuscripts. Discrepancies between the two reviewers were resolved by discussion until reached consensus among the authors. In accordance with PRISMA guidelines, the process is presented in a flow chart (15) (Figure 1).

### Data Extraction

The following data items were independently extracted by two authors (KW and TN) from the included studies; study design (author, year, and country), study population (number of included patients, age, and indication for surgery), visual analog score (VAS) for back and leg pain, Oswestry Disability Index (ODI), and parameters concerning operation (operative time, length of hospital stay, blood loss, revision) complications (total adverse events, infection, dural tear, etc.). Discrepancies were resolved by consensus.

### Quality Assessment

**Figure 2:**
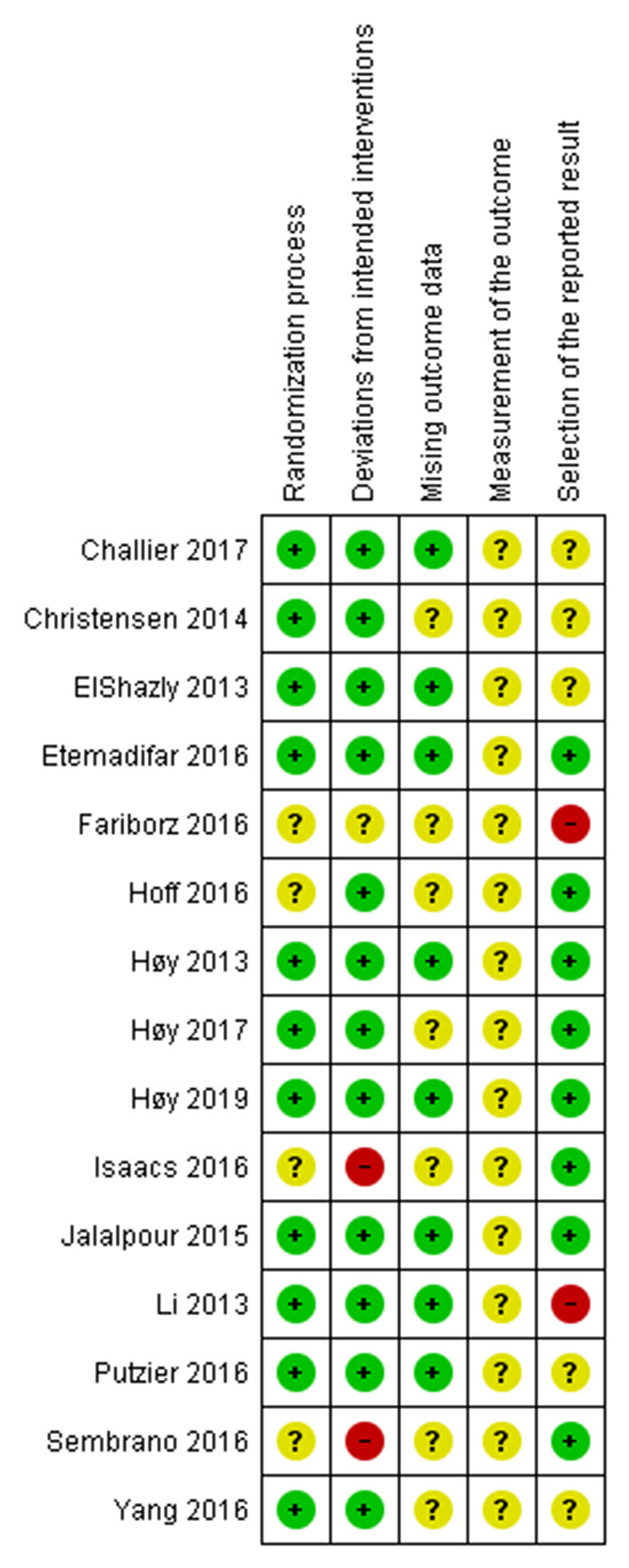
The risk of bias of each included RCT. Low risk is presented as green dot, some concerns as yellow dot, and high risk as red dot.

**Figure 3:**
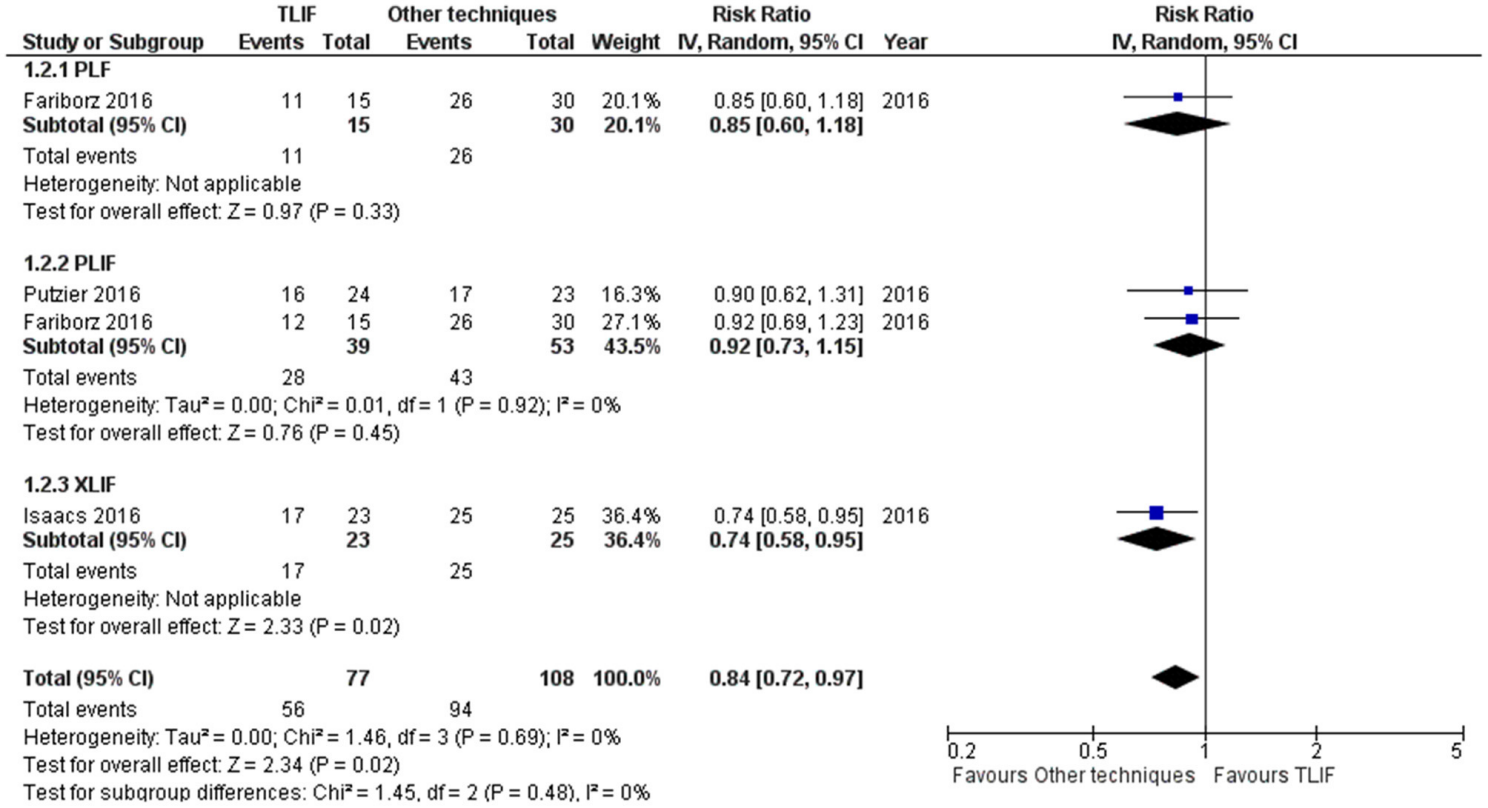
Forest plot and tabulated data illustrated the RR for fusion rate at 1 year between TLIF, PLF, PLIF, and XLIF showing that other techniques had a better arm fusion rate at 1 year and were therefore superior to TLIF in this respect. *CI*, confidence interval; *df*, degrees of freedom.

The authors worked independently to assess the risk of bias in the included trials using the Cochrane Risk of Bias tool 2.0 for an RCT study (16). We assessed the randomization process, deviations from intended intervention, missing outcome data, measurement of the outcome, and selection of the reported result. We assigned each domain as a low risk of bias, some concerns, and a high risk of bias. We contacted the authors if there was not enough information to assess. If the trial authors did not respond within 14 days, we conducted the assessment using available data. We resolved the disagreement through discussion. We presented our risk of bias assessment in Figures 2, 3.

### Statistical Analysis

The primary outcome was the fusion rate. The clinical outcomes measured were the mean difference for VAS back and leg pain, Oswestry Disability Index (ODI) score, and parameters concerning operation (operative time, blood loss, and length of hospital stay) with an associated 95% CI. Fusion rate, total adverse events, infection rate, revision rate, and dural tear were reported as the risk ratio (RR) with 95% CI. The results of the studies were included in the meta-analysis and presented in a forest plot, which also showed statistical powers, confidence intervals, and heterogeneity. The variability within-a study and between studies was assessed by an *I*^2^ estimate of heterogeneity. We regarded the level of heterogeneity for *I*^2^ statistics as defined in Chapter 9 of the Cochrane Handbook for Systematic Reviews of Interventions: 0–40% might not be important; 30–60% may represent moderate heterogeneity; 50–90% may represent substantial heterogeneity; and 75–100% considerable heterogeneity. The random effects meta-analysis by DerSimonian and Laird method was used as clinical, methodological, and statistical heterogeneity encountered. Prespecified subgroup analyses by the type of comparators were performed. We assessed publication bias by computing each study effect size against standard error and plotted it as a funnel plot to assess asymmetry visually. The significant asymmetry indicated the possibility of publication bias or heterogeneity. The meta-analysis was performed using Revman 5.3 (Cochrane Collaboration, Oxford, UK).

### Patient and Public Involvement

It was not possible to involve patients or the public in the design, conduct, reporting, and dissemination plan of this systematic review and meta-analysis.

## Results

### Systematic Review

A systematic search identified 3,682 potential English articles, among them 1,957 were removed due to duplication. Two reviewers assessed the title and abstracts of 1,725 studies which 144 manuscripts remained for full-text assessment. Eventually, 18 RCTs were met the inclusion criteria. A number of 2 RCTs were considered the same population of the TLIF group; therefore, one study was excluded from the analysis. The studies that did not report the variation were excluded. A PRISMA diagram is shown in Figure 1.

There were 15 RCTs included with 915 patients (470 TLIF, 258 PLF, 87 PLIF, 26 ALIF, 29 XLIF, and 45 no fusion) (17–31). The TLIF group in the 2 studies was in addition to PLF. Publication years ranged from 2013 to 2019. Three studies reported outcomes at 1-year follow-up whereas the other reported at least 2-year follow-up. Study characteristics are provided in Table 1.

**Table 1:** Study characteristics.

**References**	**Surgical technique**	**TLIF, *n***	**Other techniques, *n***	**Follow-Up**
Challier et al. (17)	PLF + TLIF vs. PLF	30	30	2 y
Christensen et al. (18)	TLIF vs. PLF	51	49	1, 2 y
El Shazly et al. (19)	Discectomy + TLIF vs. no fusion	15	15	2 y
	Discectomy + TLIF vs. Discectomy + PLF	15	15	2 y
Etemadifar et al. (20)	PLF + TLIF vs. PLF	25	25	1.5, 3, 6 m, 1, 2 y
Fariborz et al. (21)	TLIF vs. PLIF	30	30	6 m, 1 y
	TLIF vs. PLF	30	30	6 m, 1 y
	TLIF vs. no fusion + instrumentation	30	30	6 m, 1 y
Hoff et al. (22)	TLIF vs. ALIF TDR	24	26	1, 3 y
Høy et al. (23)^*^	TLIF vs. PLF	51	49	1, 2 y
Høy et al. (24)^*^	TLIF vs. PLF	44	44	1, 2, 5–10 y
Høy et al. (25)^*^	TLIF vs. PLF	51	49	1, 2 y
Isaacs et al. (26)^**^	TLIF vs. XLIF	26	29	1, 2 y
Jalalpour et al. (27)	TLIF vs. PLF	68	67	1, 2 y
Li et al. (28)	TLIF vs. PLF	19	18	2–5 y
Putzier et al. (29)	TLIF vs. PLIF	24	23	1 y
Sembrano et al. (30)^**^	TLIF vs. XLIF	55	26	1 y
Yang et al. (31)	TLIF vs. PLIF	32	34	3 m, 1–2 y

* *Same sample group*,

** *same sample group*.

### Quality Assessment

For the risk of bias assessment, the included RCTs had a relatively high percentage of low risk in the randomization process and deviations from intended intervention domains. All included RCTs had some concerns risk of bias in the measurement of the outcome. There was some high risk of bias in deviations from intended interventions and selection of the reported result domains. Detailed risk-of-bias assessment for included RCTs is provided in Figure 2. A summary of the percentages of RCTs which were at low, some concerns, and high risk for each risk of bias domain was presented in Supplementary File 1. The funnel plots showed no significant asymmetry which highlighted no evidence of publication bias on the fusion rate, total adverse events, and revision rate (Supplementary File 1).

### Meta-Analysis

A total of 15 included studies were included in the meta-analysis with 915 patients (470 TLIF, 258 PLF, 87 PLIF, 26 ALIF, 29 XLIF, and 45 no fusion).

### Fusion Rate

Fusion rate was 72.7% on TLIF group at 1-year follow-up whereas 87.03% fusion rate was reported on other techniques [PLF, PLIF, XLIF; 4 studies]. TLIF had slightly lower fusion rate at 1-year follow-up compared to other techniques [PLF, PLIF, XLIF; 5 studies] [RR = 0.84 (95% CI = 0.72–0.97), *p* = 0.02, *I*^2^ = 0.0%] (Figure 3). However, the fusion rate at 2 years did not show any statistically significant differences [RR = 1.06 (95% CI = 0.96–1.18), *p* = 0.27, *I*^2^ = 69.0%] as shown in Supplementary File 1.

### Complications: Total Adverse Events, Revision, Infection, and Dural Tear

**Figure 4:**
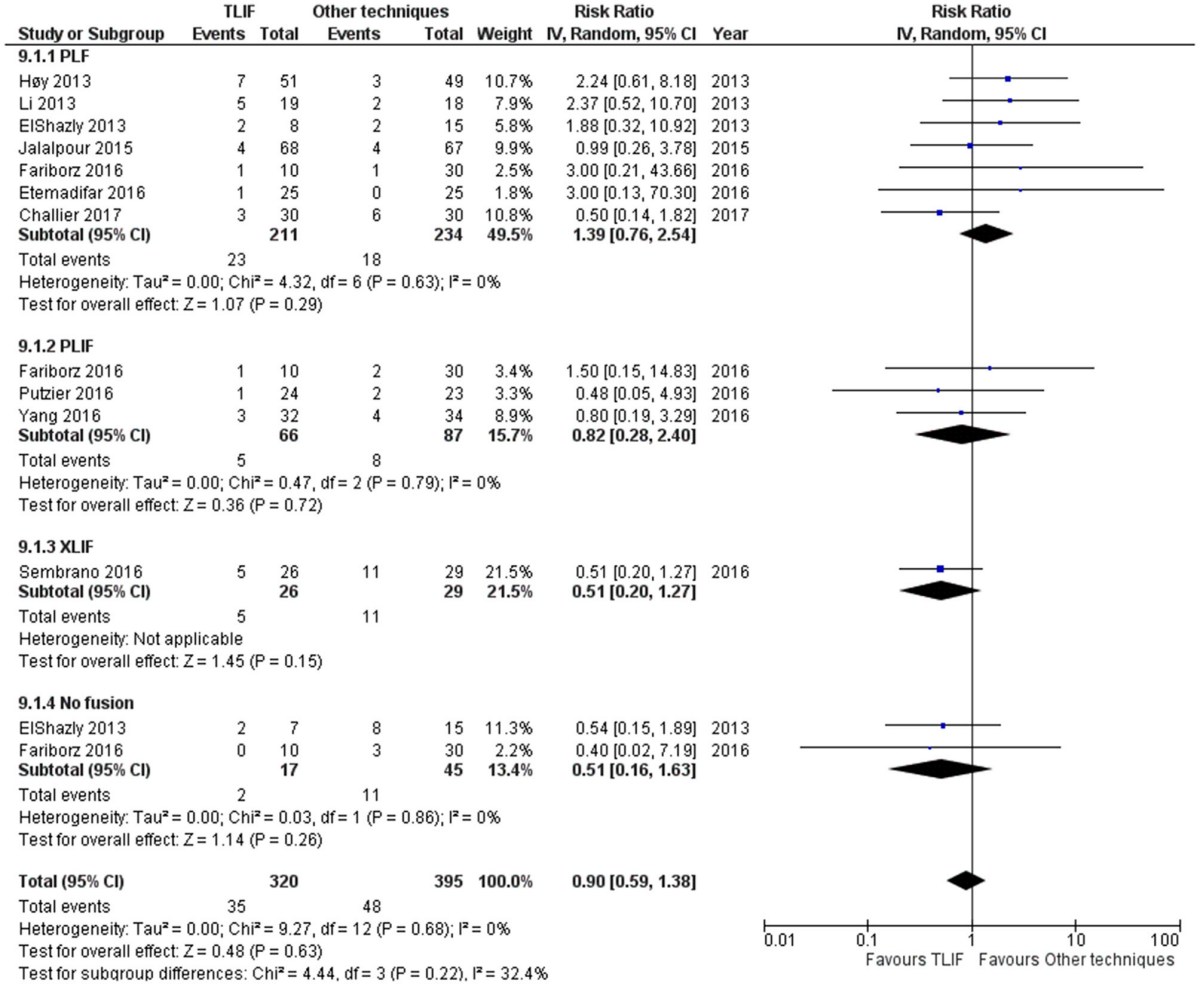
Forest plot and tabulated data illustrated the RR for adverse events between TLIF, PLF, PLIF, XLIF, and no fusion showing that there was no significant difference in adverse events between procedures. *CI*, confidence interval; *df*, degrees of freedom.

Total adverse events were reported in 10 studies. TLIF had similar total adverse events compared with PLIF, XLIF, and no fusion group [RR = 0.90 (95% CI = 0.59–1.38), *p* = 0.63, *I*^2^ = 0.0%] as shown in Figure 4. For the revision needed after surgical procedures, the results indicated a different revision rate among groups [no fusion, PLF, PLIF, XLIF] [RR = 0.78 (95% CI 0.34–1.79), *p* = 0.56, *I*^2^ = 39.0%] as shown in Supplementary File 1.

Infection was reported in 6 studies [no fusion, PLF, PLIF], and overall infection was similar among groups [RR = 1.78 (95% CI 0.58–5.46), *p* = 0.31, *I*^2^ = 0.0%]. More infection was reported in the TLIF group but was not statistically significant. The dural tear was higher in other techniques especially XLIF group but not statistically significant [RR = 1.19 (95% CI = 0.49–2.89), *p* = 0.70, *I*^2^ = 0.0%]. The results of secondary outcomes were reported as shown in Table 2.

**Table 2:** Secondary outcomes.

**Outcomes**	**Studies**	**Patients**	**Statistical method**	**Effect size [95% CI]**
Infection	6	433	IV, random, 95% CI	RR = 1.78 [95% CI = 0.58–5.46], *p* = 0.31, *I^2^* = 0.0%
Dural tear	7	570	IV, random, 95% CI	RR = 1.19 [95% CI = 0.49–2.89], *p* = 0.70, *I^2^* = 0.0%
Operative time	6	353	IV, random, 95% CI	MD = 31.88 [95% CI = 5.33–58.44], *p* = 0.02, *I^2^* = 92.0%
Blood loss	4	248	IV, random, 95% CI	191.00 [95% CI = −53.93–435.93], *p* = 0.13, *I^2^* = 90.0%
Length of hospital stay	3	200	IV, random, 95% CI	MD = 0.12 [95% CI = −0.30–0.54], *p* = 0.58, *I^2^* = 0.0%
VAS back at last follow-up	6	335	IV, random, 95% CI	MD = 0.13 [95% CI = −0.40–0.66], *p* = 0.62, *I^2^* = 82.0%
VAS leg at last follow-up	2	150	IV, random, 95% CI	MD = −0.07 [95% CI = −1.43–1.30], *p* = 0.92, *I^2^* = 77.0%
ODI at last follow-up	7	521	IV, random, 95% CI	MD = −4.82 [95% CI = −11.72–2.08], *p* = 0.17, *I^2^* = 90.0%

*CI, confidence interval; IV, inverse variance; MD, mean difference; RR, risk ratio; VAS, visual analog scale*.

### Operative Time

Anterior lumbar interbody fusion, PLF, and no fusion groups have shorter operative time whereas PLIF has longer operative time compared to TLIF. The pooled mean difference in operative time of other techniques was 31.88 min shorter than TLIF [no fusion, ALIF, PLF, PLIF, XLIF: 7 studies] [MD = 31.88 (95% CI = 5.33–58.44), *p* = 0.02, *I*^2^ = 92.0%].

### Blood Loss

Transforaminal lumbar interbody fusion has less blood loss than PLIF 88.80 ml. No fusion has less blood loss among groups [no fusion, PLF, PLIF: 5 studies]. Pooled mean difference in blood loss showed no significant difference [MD = 191.00 (95% CI −53.93–435.93), *p* = 0.13, *I*^2^ = 90.0%].

### Length of Hospital Stay

Length of hospital stay between subgroup was not significantly different. Pooled mean difference in hospital stay was 0.12 [MD = 0.12 (95% CI = −0.30–0.54), *p* = 0.58, *I*^2^ = 0.0%] [no fusion, PLF, XLIF: 4 studies].

### Back and Leg Pain

Visual analog scale (VAS) for back were extracted from 7 studies. There was no difference between back pain at last follow-up in TLIF and other technique groups [MD = 0.13 (95% CI = −0.40–0.66), *p* = 0.62, *I*^2^ = 82.0%] [no fusion, ALIF, PLF, PLIF: 7]. ALIF [MD = 1.20 (95% CI = 0.53–1.87), *p* < 0.01] and no fusion techniques [MD = 0.60 (95% CI = 0.08–1.12), *p* = 0.02] were shown less back pain at last follow-up. VAS for leg was extracted from only 2 PLF studies. There was no difference between leg pain at last follow-up in TLIF and PLF groups [MD = −0.07 (95% CI = −1.43–1.30), *p* = 0.92, *I*^2^ = 77.0%].

### ODI

No difference in ODI was observed [MD = −4.82 (95% CI = −11.72–2.08), *p* = 0.17, *I*^2^ = 90.0%]. Compared to TLIF, no fusion group had higher ODI at last follow-up [MD = −41.30 (95% CI = −5–0.15–−32.45), *p* < 0.001] [no fusion, ALIF, PLF, PLIF: 9 studies].

## Discussion

Patients with degenerative lumbar spine disease require surgical intervention when the conservative treatments failed (7–10). The operative methods are varyingly selected among spine surgeons. Therefore, the fusion rates and other clinical outcomes were reported in different studies. This systematic review and meta-analysis attempted to investigate the benefits and risks of lumbar interbody fusion, no fusion, and posterolateral fusion by comparing the fusion rate, clinical outcomes, and parameters concerning operation, as well as complications.

The surgical techniques of PLIF vary across studies whereas one study did not mention the details of the surgical procedure (23). Putzier et al. used the conventional five centimeters midline approach and place a cage bilaterally (29). Yang et al. performed PLIF in the standard fashion with two rectangular cages packed with autogenous bone grafts (31). The ALIF procedure uses a pararectal retroperitoneal approach. A stand-alone PEEK cage filled with freeze-dried allogenic cancellous bone was fixed with four angle-stable screws at L5-S1 and a prosthesis at L5-S1 (24). The XLIF utilizes a mini-open, 90° off-midline, retroperitoneal, trans-psoas approach for ALIF. Two RCTs (26, 30) included in this study had equal sample sizes. The authors described using the same technique that was previously described (5, 32). Direct decompression was not performed in XLIF patients.

Of the three RCTs on MIS-TLIF (26, 29, 30), two were open TLIF (22, 27) and the other did not specify surgical details of TLIF. We compared the clinical outcomes and complications among operative techniques; therefore, we included MIS-TLIF, open TLIF, and no details mentioned TLIF as a TLIF group. Nonetheless, MIS-TLIF and open TLIF have demonstrated similar clinical outcomes (33–35) whereas the comparison of open TLIF vs. MIS-TLIF is beyond the scope of this study.

From the currently available evidence, findings from our study were similar to the previous systematic review that reported an 89.1% fusion rate and 12.5% reoperation rate (36). Manzur et al. reported an 85.6% fusion rate on LLIF (37). The evidence that supports a higher fusion rate compared with TLIF was rare (10). Lan's et al. study in which PLIF compared with TLIF demonstrated similar outcomes (11). The TLIF has a slightly low fusion rate at 1 year and remains unchanged at 2 years. Further study on potential multifactorial factors supports the fusion (9).

In terms of pain, there was less back pain in the non-fusion group and similar back pain among fusion groups. In no fusion group, the surgery was less invasive compared to the fusion group, therefore resulting in less back pain. In pooled outcome data, there was no significant difference in ODI scores between surgical techniques. At the last follow-up, the no fusion group had a higher ODI score compared to the TLIF group. As time passes, patients in the no fusion group may result in higher disability. Less paravertebral dissection of no fusion group affected in less operative time and blood loss. PLIF has the longest operative time when compared to no fusion, PLF, ALIF, and TLIF. PLIF has also more blood loss than TLIF. This might result from a posterior approach in which more paravertebral muscle was dissected and the bone structure was resected more than TLIF (14).

Surgical complications evaluated by total adverse events were not shown statistically significant differences among lumbar interbody fusion, no fusion, and posterolateral fusion. However, our result TLIF seems to be safer than PLIF and ALIF in neural, spinal, and vascular events. Those findings were similar to a previous study by Chi et al. (9). Nonetheless, Yavin et al. demonstrated more complications in the fusion group compared to the non-fusion group (38).

The strength of this study was that we included only RCTs that showed no significant asymmetry that highlighted no evidence of publication bias on the fusion rate, total adverse events, and revision rate. However, the small number of RCT on TLIF was the limitation of our study. The heterogeneity of the enrolled studies was another limitation. The study (19) with the procedure of TLIF + discectomy was counted as the TLIF group. Furthermore, there was the same sample group in three studies as shown in Table 1 (23–25). We try to reduce the bias by excluding the repeated data from the analysis. For lumbar spine disease, the included studies were different in treatment protocol which may affect the results, for example, fusion rate (because not all studies have reported outcomes at 2-year follow-up). Furthermore, the results are referred to only single-level surgery as the included studies.

## Conclusion

Besides fusion rate at 1-year follow-up and operative time, TLIF has a similar fusion rate, clinical outcomes, parameters concerning operation and complications to decompression alone (no fusion), posterolateral fusion, and other interbody fusion (PLIF, ALIF, and XLIF).

## Data Availability Statement

The original contributions presented in the study are included in the article/Supplementary Material, further inquiries can be directed to the corresponding author/s.

## Author Contributions

KW and KP contributed to the study conception and design. Material preparation and data collection were performed by KW and TN. Data analysis was performed by TN. All authors wrote the first draft of the manuscript and revised and approved the final manuscript.

## Conflict of Interest

The authors declare that the research was conducted in the absence of any commercial or financial relationships that could be construed as a potential conflict of interest.

## Publisher's Note

All claims expressed in this article are solely those of the authors and do not necessarily represent those of their affiliated organizations, or those of the publisher, the editors and the reviewers. Any product that may be evaluated in this article, or claim that may be made by its manufacturer, is not guaranteed or endorsed by the publisher.
